# Characteristic cytokine profile of the aqueous humor in eyes with congenital cataract and pre-existing posterior capsule dysfunction

**DOI:** 10.3389/fmed.2024.1301588

**Published:** 2024-02-16

**Authors:** Yinying Zhao, Qihui Zhao, Hongfang Zhang, Zhewen Zhang, Dandan Wang, Zhangliang Li, Xixia Ding, Yune Zhao

**Affiliations:** ^1^The School of Ophthalmology and Optometry, Wenzhou Medical University, Wenzhou, Zhejiang, China; ^2^National Clinical Research Center for Ocular Diseases, Wenzhou, Zhejiang, China

**Keywords:** cytokine profile, aqueous humor, congenital cataract, posterior capsule dysfunction, inflammatory factors

## Abstract

**Objectives:**

To investigate the characteristic cytokine profile of the aqueous humor in eyes with congenital cataract and pre-existing posterior capsule dysfunction (PCD).

**Methods:**

In this cross-sectional study, the enrolled eyes with congenital cataract and PCD were included in the PCD group, while those with an intact posterior capsule were included in the control group. Demographic data and biometric parameters were recorded. The levels of 17 inflammatory factors in the aqueous humor collected from the enrolled eyes were detected using Luminex xMAP technology, and intergroup differences in the collected data were analyzed.

**Results:**

The PCD group comprised 41 eyes from 31 patients with congenital cataract and PCD, whereas the control group comprised 42 eyes from 27 patients with congenital cataract and an intact posterior capsule. Lens thickness was significantly thinner in the PCD group than in the control group. However, the levels of monocyte chemoattractant protein-1 (MCP-1), transforming growth factor-β2 (TGF-β2), and vascular endothelial growth factor (VEGF) were significantly higher in the PCD group than in the control group. Multivariate logistic regression confirmed that lens thickness and TGF-β2 level were independent risk factors for PCD.

**Conclusion:**

A thinner lens thickness in eyes with congenital cataract and PCD could serve as a biometric feature of these eyes. The higher levels of MCP-1, TGF-β2, and VEGF in eyes with PCD indicated a change in their intraocular inflammatory microenvironment, which possibly led to cataract progression. Lens thickness and TGF-β2 level are independent risk factors for PCD.

## Introduction

Congenital cataract is the most common cause of treatable childhood blindness worldwide, and it has a prevalence as high as 1.7–14.7/10,000 children in Asia (1, 2). Various concurrent ocular and systemic abnormalities are sometimes observed in patients with congenital cataract, including pre-existing posterior capsule dysfunction (PCD) (3, 4).

The classic signs of PCD are demarcated, thick defect margins and white dots on the posterior capsule and anterior vitreous (3). PCD has been reported as a rare concurrent abnormality of congenital cataract in recent years. However, owing to the occult nature of PCD, it is often ignored by clinicians. Therefore, the incidence rate of PCD is underestimated in patients with congenital cataract. Our previous study showed that the percentage of PCD in eyes with congenital cataract was as high as 26.7% (5). The presence of PCD is probably the cause of progressive mature cataract in children, but the underlying mechanism remains unknown (6).

Disorders of the intraocular inflammatory microenvironment are widely observed in patients with ocular diseases (7, 8). As PCD disrupts the barrier between the lens and the aqueous and vitreous humors, the cytokine profile of the aqueous humor undergoes hitherto unknown changes. In this study, we aimed to investigate the characteristic cytokine profile of the aqueous humor collected from eyes with congenital cataract and PCD in order to attempt to explain the role of cytokines in the progression of congenital cataract.

## Methods

This cross-sectional study was approved by the Institutional Ethics Review Board of the eye hospital affiliated Wenzhou Medical University (approval no. S2020-100-K87-01). The study was conducted in compliance with the Declaration of Helsinki. Informed consent was obtained from the legal guardians of all the enrolled patients. The study was registered at www.clinicaltrials.gov, under the clinical trial accession number NCT04120818.

### Patients

Patients who were diagnosed with congenital cataract by an experienced ophthalmologist and who underwent congenital cataract surgery at the Eye Hospital of Wenzhou Medical University (Hangzhou Branch) were considered eligible for this study. The exclusion criterion was the presence of ophthalmic diseases with a special intraocular inflammatory status, such as glaucoma and uveitis. PCD was diagnosed during surgery and after cataract removal. Among the enrolled eyes, those with PCD were included in the PCD group, while those with an intact posterior capsule were included in the control group.

### Data collection

Demographic data, including sex, age, and laterality of the enrolled eyes, were recorded. The following biometric parameters were measured under sedation. Intraocular pressure (IOP) was measured using a handheld tonometer (Icare Finland Oy, Vantaa, Finland). Axial length (AL), lens thickness (LT), and vitreous chamber depth (VCD) were measured using a contact A-scan (Axis Nano, Quantel Medical). Horizontal corneal diameter (CH) and vertical corneal diameter (CV) were measured using calipers during surgery. The K values were measured using a handheld keratometer (HandyRef-K, Nidek Co., Ltd). For each patient, the K values were measured and A-scans were performed three times, while IOP was measured six times. The mean values were recorded.

### Aqueous humor sample collection

Aqueous humor samples were collected using a 27-gauge needle via limbal paracentesis at the beginning of the surgery before any incisional procedures. Care was taken to avoid touching the iris, lens, or corneal endothelium. A total of 50–200 μL of aqueous humor was gently withdrawn per eye, transferred into aseptic EP tubes, and immediately stored at −80°C until analysis.

### Measurement of cytokine levels

The levels of 17 cytokines in 80 samples were measured, including those of interleukin (IL)-1, IL-1A, IL-2, IL-4, IL-5, IL-6, IL-10, IL-12, IL-15, IL-17A, interferon γ-induced protein 10 (IP-10), interferon (IFN), monocyte chemoattractant protein-1 (MCP-1), tumor necrosis factor (TNF), transforming growth factor-β2 (TGF-β2), granulocyte colony-stimulating factor (G-CSF), and vascular endothelial growth factor (VEGF). The levels were detected using Luminex xMAP technology with multi-analyte profiling beads (Lincoplex cytokine/chemokine multiplex kit, HCYTO-60 K; Millipore Corporation, Billerica, MA). The multiplexed microsphere-based immunoassays were used combined with flow cytometric resolution to measure microspheres coupled with fluorochromes combined to detection antibodies. The detailed measurement procedures have been described in previous study (9, 10).

### Statistical analysis

The data were analyzed using IBM SPSS Statistics for Windows/Macintosh, Version 22.0 (IBM Corp., Armonk, NY). The Shapiro–Wilk test was used to evaluate the normality of the distribution for all variables. For values fitting the normal distribution, comparisons between the PCD and control groups were conducted using the independent-samples t-test; otherwise, the nonparametric Mann–Whitney U test and Kruskal–Wallis H test were used. Multivariate logistic regression was used to assess the association between the parameters and PCD. A two-tailed *p* value less than 0.05 was considered statistically significant for all tests.

### Patient and public involvement

No patient or member of the public was involved in either the design, or conduct, or reporting, or dissemination plans of this research.

## Results

### Demographic data of the enrolled patients

The PCD group comprised 41 eyes from 31 patients with congenital cataract and PCD, while the control group comprised 42 eyes from 27 patients with congenital cataract and an intact posterior capsule. Their demographic data are summarized in Table 1. No significant differences were observed between the two groups.

**Table 1:** Demographic data of the enrolled patients.

Characteristics	PCD group	Control group	*P*-value
Age	5.24 ± 2.45	4.50 ± 1.95	0.189
Gender			0.122
Male	13 (41.9%)	17 (63.0%)	
Female	18 (58.1%)	10 (37.0%)	
Laterality			
Unilateral	21 (67.7%)	12 (44.4%)	
Bilateral	10 (32.3%)	15 (55.6%)	

### Intergroup comparisons of preoperative biometric parameters

The values of preoperative biometric parameters are presented as the mean ± standard deviation and are summarized in Table 2. LT was significantly thinner in eyes with congenital cataract and PCD than in those with an intact posterior capsule (*p* = 0.013). No significant intergroup differences were observed in IOP, AL, VCD, CH, CV, K1, and K2.

**Table 2:** Preoperative biometric parameters of two groups (mean ± standard deviation).

Parameters	PCD group	Control group	*P*-value
IOP (mmHg)	11.7 ± 2.09	12.37 ± 2.11	0.176
AL (mm)	18.5 ± 1.32	18.93 ± 1.36	0.255
LT (mm)	3.11 ± 0.95	3.69 ± 1.08	0.013*
VCD (mm)	12.91 ± 1.25	12.7 ± 1.73	0.365
CH (mm)	9.54 ± 0.64	9.77 ± 0.55	0.087
CV (mm)	9.13 ± 0.64	9.31 ± 0.59	0.101
K1 (D)	42.56 ± 2.21	42.73 ± 2.42	0.756
K2 (D)	45.02 ± 2.48	44.8 ± 2.6	0.984

Independent-samples *t-*test. *P < 0.05. IOP, intraocular pressure; AL, axial length; LT, lens thickness; VCD, vitreous chamber depth; CH, horizonal corneal diameter; CV, vertical corneal diameter; K1, keratometry 1; K2, keratometry 2.

### Intergroup differences in the levels of cytokines

**Figure 1:**
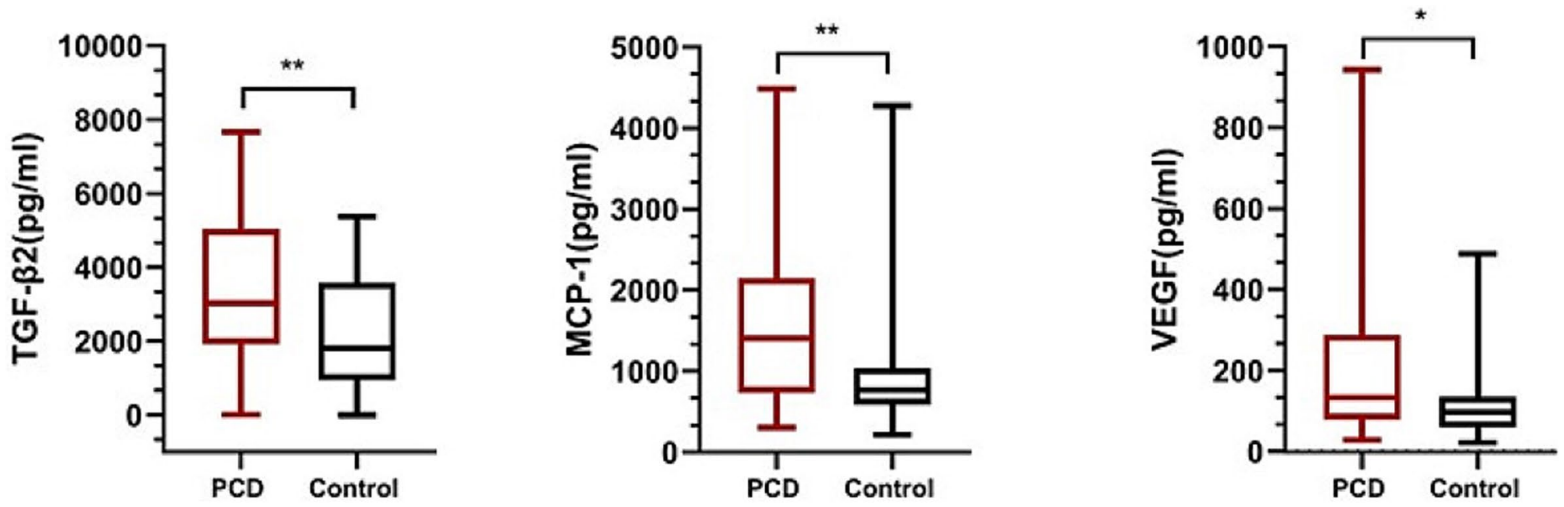
The level of TGF-β2, MCP-1, VEGF in posterior capsule dysfunction (PCD) group and control group. **p < 0.01; *p < 0.05.

The levels of IL-1, IL-1A, IL-2, IL-4, IL-5, IL-6, IL-10, IL-12, IL-15, IL-17A, IP-10, IFN, MCP-1, TNF, TGF-β2, G-CSF, and VEGF in 80 aqueous humor samples are summarized as the median and 25th to 75th interquartile range in Table 3. The level of MCP-1 in eyes with congenital cataract and PCD was higher than that in eyes with an intact posterior capsule (*p* = 0.001), as were the levels of TGF-β2 (*p* = 0.003) and VEGF (*p* = 0.04) (Figure 1). No intergroup differences were observed in the levels of the other detected cytokines.

**Table 3:** The concentrations (pg/mL) of cytokines in PCD group and control group.

	PCD group			Control group			*P*-value
	Mdn	Q25	Q75	Mdn	Q25	Q75	
IL-1	40.61	37.80	42.95	40.80	36.38	44.87	0.340
IL-1A	188.04	120.25	650.22	209.60	117.02	301.83	0.359
IL-2	192.15	1.21	301.71	177.10	0.43	266.61	0.186
IL-4	31.89	0.31	36.22	21.50	0.19	34.22	0.277
IL-5	3.34	3.12	3.54	3.36	3.10	3.39	0.607
IL-6	6.08	3.59	19.77	5.50	2.98	12.47	0.207
IL-10	1.20	1.03	92.59	1.85	0.62	89.10	0.506
IL-12	100.17	89.12	104.06	92.44	86.82	110.65	0.249
IL-15	7.33	2.69	7.96	4.30	1.71	5.34	0.163
IL-17A	10.32	7.52	11.46	6.46	6.01	17.59	0.270
IP-10	45.05	11.49	107.96	50.11	33.51	89.94	0.328
IFN	40.61	37.80	42.95	40.80	36.38	44.87	0.613
MCP-1	1404.25	734.90	2148.24	767.40	583.78	1039.74	0.001*
TNF	3.45	2.21	3.82	2.53	0.87	3.34	0.113
TGF-β2	3187.43	2024.61	5119.51	1808.76	951.66	3579.52	0.003*
G-CSF	11.64	9.19	12.62	11.12	6.13	12.46	0.258
VEGF	132.92	78.96	288.54	97.20	59.30	135.61	0.040*

Mann–Whitney U test. *P < 0.05.

### Differences in the levels of MCP-1, TGF-β2, and VEGF among the three types of PCD

**Figure 2:**
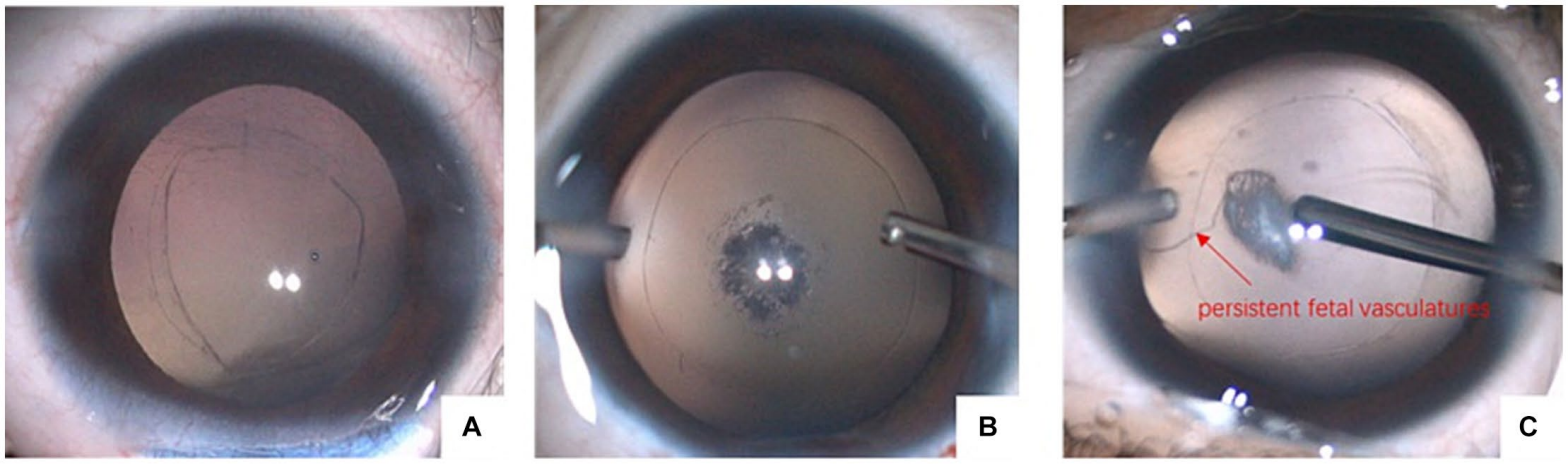
Three types of PCD. **(A)** Large defect with sinking cortex in anterior vitreous; **(B)** reticular defect or cluster of fibrotic spots in posterior capsule; **(C)** posterior capsule defect with concurrent PFV.

We further divided the eyes with PCD into three types, as shown in Figure 2. Type I was characterized by a large defect with a “sinking cortex” sign in the anterior vitreous; Type II was characterized by a reticular defect or cluster of fibrotic spots in the posterior capsule; and Type III was characterized by a posterior capsule defect with concurrent persistent fetal vasculature (PFV). The number of eyes with the cytokine levels in the three types of PCD are summarized in Table 4. No significant differences were observed in the levels of MCP-1, TGF-β2, and VEGF among the three types.

**Table 4:** The concentrations (pg/mL) of MCP-1, TGF-β2, and VEGF in three types of PCD and control groups as median (25th, 75th).

	Type I	Type II	Type III	Control group	*P*-value
N (Eyes)	18	14	9		
MCP-1	1530.30 (620.12, 2473.32)	1425.09 (934.90, 2030.85)	1147.51 (730.60, 2154.90)	767.40(583.78, 1039.74)	0.340
TGF-β2	3357.62 (2425.43, 4511.84)	2540.45 (1059.82, 5753.14)	2446.96 (1810.64, 4947.94)	1808.76 (951.66, 3579.52)	0.359
VEGF	87.24 (51.75, 284.70)	213.15 (82.93, 301.33)	132.93 (95.66, 318.49)	97.20 (59.30, 135.61)	0.186

Type I: large defect with sinking cortex in anterior vitreous. Type II: reticular defect or cluster of fibrotic spots in posterior capsule. Type III: posterior capsule defect with concurrent PFV. The Kruskal-Wallis test demonstrated that the differences of MCP-1, TGF-β2, and VEGF levels in three types of PCD were no statistically significant.

### Multivariate analysis for associations between the preoperative predictors and PCD

LT, MCP-1, TGF-β2, and VEGF were significantly associated with PCD in the univariate analysis. The results of the multivariate logistic regression indicated that preoperative LT (*p* = 0.009) and the level of TGF-β2 (*p* = 0.027) were independent risk factors for PCD.

## Discussion

As the major cause of treatable childhood blindness worldwide, congenital cataract has drawn widespread attention from surgeons and researchers. Nevertheless, many features, such as the exact etiology and pathogenesis of congenital cataract, remain unexplored. In recent years, PCD has been considered a form of concurrent abnormality of congenital cataract. Li divided PCD into the following three types: Type I, which is characterized by a large defect with a “sinking cortex” sign in the anterior vitreous, has also been reported as a posterior capsule defect by Vajpayee and has been described as a sign of demarcated, thick defect margins and white dots on the posterior capsule and in the anterior vitreous by Vasavada (3, 11); Type II is characterized by a reticular defect or a cluster of fibrotic spots in the posterior capsule; and Type III is characterized by a posterior capsule defect with concurrent PFV (12). These types probably indicate that PCD in children shows a dynamic progression.

Meanwhile, because PCD disrupts the barrier between the lens and the aqueous and vitreous humors, the exposed cortex might lead to a change in the intraocular inflammatory environment, which in turn might cause the dynamic progression of PCD. Moreover, PCD seems to be the cause of progressive mature cataract in children, as reported by Vasavada et al. (6). However, no study to date has definitively demonstrated the mechanism underlying PCD and related cataract. Our previous study confirmed that LT in eyes with congenital cataract and PCD was thinner than that in eyes without PCD (12). The same result was also obtained in the current study. These findings suggested that a thinner LT could serve as a characteristic biometric feature of PCD in eyes with congenital cataract. In contrast to eyes with PCD, those with traumatic cataract resulting from capsule rupture had a greater LT. Moreover, the existence of PCD in infants with congenital cataract likely influenced the formation of crystalline lens fibers or resulted in the dissolution of the lens cortex. However, this hypothesis is not yet supported by any published study.

The lens capsule, whose main structural components are collagen type IV, laminin, nidogen, and heparan sulfate proteoglycans, is a relatively thick basement membrane completely encasing the cells of the lens, thus sequestering them from the surrounding aqueous and vitreous humors (13). The anterior capsule mainly consists of collagen secreted by the lens epithelial cells and is thus thicker, whereas the posterior capsule consists of the secretions of lens fibroblasts (14).

Müllner-Eidenböck et al. hypothesized that all cases of unilateral congenital cataract were associated with PFV, thereby implying that the incidence of PFV was significantly higher in unilateral congenital cataract than in other types of cataract (15). Similarly, the incidence of posterior capsule plaques was also higher in unilateral congenital cataract (16). Therefore, the Infant Aphakic Treatment Study Group hypothesized that posterior capsule plaques might represent a mild form of PFV. In our previous study, we hypothesized that PCD might result from PFV if the fetal vessels had passed through the posterior capsule and thus made it weak (5).

An argument against this hypothesis is that an abnormal vitreo-lenticular interface is the primary cause of PCD. Proteomic analysis revealed that collagen type IV, the main structural component of the lens capsule, was not observed in any of the posterior capsule plaques (17). Another study speculated that the presence of an abnormal vitreo-lenticular interface led to the pathophysiologic pathway of posterior capsule plaques (18).

The intraocular inflammatory microenvironment has been widely investigated in other ocular diseases, such as high myopia, glaucoma, and uveitis, but not in PCD. Because PCD disrupts the barrier between the lens and the aqueous and vitreous humors, the exposed cortex might lead to changes in the intraocular inflammatory microenvironment. MCP-1 is a biomarker of inflammation that regulates the migration of monocytes/macrophages (19). The high levels of MCP-1 observed in eyes with congenital cataract and PCD in our study proved our hypothesis that the intraocular inflammatory microenvironment changes as PCD disrupts the barrier between the lens and the aqueous and vitreous humors. Previous studies have also demonstrated that the level of MCP-1 is upregulated in patients with retinitis pigmentosa and high myopic cataract, thereby suggesting a proinflammatory status in these eyes (20, 21). Our observation of higher levels of MCP-1 in the PCD group rather than in the control group also indicated a proinflammatory status in eyes with PCD. However, the level of IL-6, which is another macrophage-derived cytokine, did not show a significant change in our study groups. Therefore, MCP-1 and IL-6 likely play different roles at different stages of the inflammatory response induced by PCD.

The change in the intraocular inflammatory microenvironment might potentially stabilize the repair process of the posterior capsule, and this might explain why the vast majority of cytokines in our study did not show significant changes as PCD could have been repaired somehow and could have prevented persistent cortex exposure. As the major isoform of TGF-β (22), TGF-β2 is known to be the key growth factor in the repair of damaged tissue. TGF-β2 plays a major role in ocular scar formation by inducing the expression of collagen type IV and promoting cell proliferation in human Tenon’s fibroblasts after glaucoma filtration surgery (23, 24). In our study, the level of TGF-β2 was higher in eyes with PCD than in those without PCD. Therefore, we hypothesized that the presence of PCD stimulated the repair of the posterior capsule. As a result, the level of TGF-β2 was abnormally upregulated. This abnormal upregulation then aberrantly induced incomplete repair, leading to a reticular defect, posterior capsule contraction, and posterior capsule plaque. We divided PCD into three types. Even though, we could not find any difference TGF-β2 between the three types of PCD, it was higher in PCD of type I compared with type II and type III. We found the difference of the exposed cortex may lead to the different level of TGF-β2. The large defect of type I with cortex exposure in aqueous humors induced more TGF-β2, while type II and type III with tiny defect started self-repairment induced less TGF-β2. Further studies are warranted to investigate the underlying mechanisms.

In the present study, higher levels of VEGF were detected in the aqueous humor samples collected from eyes with congenital cataract and PCD than in those without PCD. Zhang et al. provided evidence that astrocytes in the retained vessels of patients with PFV express high levels of VEGF (25). The higher level of VEGF in eyes with PCD seemed reasonable if the posterior capsule plaque represented a mild form of PFV as previously hypothesized. However, more cases of PCD without PFV or minimal PFV are encountered in a clinical setting. Moreover, no significant difference was observed in the levels of VEGF among the three subgroups irrespective of the presence of PFV. Taken together, these observations suggest that PFV may play a role in PCD in children, even though the vasculature was not found.

To the best of our knowledge, this is the first study to investigate the characteristic cytokine profiles of the aqueous humor in eyes with congenital cataract and PCD and to speculate on the underlying mechanism of the progression from PCD to cataract. Nevertheless, the small sample size was a limitation of this study, as only 41 and 42 eyes could be enrolled into the PCD and control groups, respectively. Another limitation was that we could not reveal the mechanism underlying PCD formation, and further studies on embryonic development may be required to unravel these processes.

## Conclusion

In conclusion, this study investigated the differences between eyes with congenital cataract with and without PCD in terms of biometric parameters and cytokine levels in the aqueous humor. LT was significantly thinner in eyes with PCD, and this served as the biometric feature of PCD. The levels of MCP-1, TGF-β2, and VEGF were also higher in eyes with PCD. Changes in the intraocular inflammatory microenvironment in eyes with PCD potentially play a role in the progression from PCD to cataract, but further studies are warranted to determine the specific mechanism.

## Data availability statement

The original contributions presented in the study are included in the article/supplementary material, further inquiries can be directed to the corresponding author.

## Ethics statement

The studies involving humans were approved by the Institutional Ethics Review Board of the Eye Hospital Affiliated Wenzhou Medical University. The studies were conducted in accordance with the local legislation and institutional requirements. Written informed consent for participation in this study was provided by the participants’ legal guardians/next of kin. Written informed consent was obtained from the individual(s), and minor(s)’ legal guardian/next of kin, for the publication of any potentially identifiable images or data included in this article.

## Author contributions

YiZ: Conceptualization, Formal analysis, Writing – review & editing. QZ: Data curation, Writing – original draft. HZ: Data curation, Writing – original draft. ZZ: Data curation, Writing – original draft. DW: Writing – original draft. ZL: Writing – review & editing. XD: Data curation, Writing – review & editing. YuZ: Conceptualization, Funding acquisition, Writing – review & editing.
